# Persistence of Adverse Drug Reaction-Related Hospitalization Risk Following Discharge

**DOI:** 10.3390/ijerph19095585

**Published:** 2022-05-04

**Authors:** Olive Schmid, Bonnie Bereznicki, Gregory Mark Peterson, Jim Stankovich, Luke Bereznicki

**Affiliations:** 1 School of Pharmacy and Pharmacology, University of Tasmania, Hobart, TAS 7001, Australia; g.peterson@utas.edu.au (G.M.P.); jim.stankovich@gmail.com (J.S.); luke.bereznicki@utas.edu.au (L.B.); 2 Tasmanian School of Medicine, University of Tasmania, Hobart, TAS 7000, Australia; bonnie.bereznicki@utas.edu.au

**Keywords:** adverse drug reactions, medication errors, medication safety, adverse drug event, hospital admission, risk factors

## Abstract

This retrospective cohort study analyzed the administrative hospital records of 91,500 patients with the aim of assessing adverse drug reaction (ADR)-related hospital admission risk after discharge from ADR and non-ADR-related admission. Patients aged ≥18 years with an acute admission to public hospitals in Tasmania, Australia between 2011 and 2015 were followed until May 2017. The index admissions (*n* = 91,550) were stratified based on whether they were ADR-related (*n* = 2843, 3.1%) or non-ADR-related (*n* = 88,707, 96.9%). Survival analysis assessed the post-index ADR-related admission risk using (1) the full dataset, and (2) a matched subset of patients using a propensity score analysis. Logistic regression was used to identify the risk factors for ADR-related admissions within 90 days of post-index discharge. The patients with an ADR-related index admission were almost five times more likely to experience another ADR-related admission within 90 days (*p* < 0.001). An increased risk persisted for at least 5 years (*p* < 0.001), which was substantially longer than previously reported. From the matched subset of patients, the risk of ADR-related admission within 90 and 365 days more than doubled in the patients with an ADR-related index admission (*p* < 0.0001). These admissions were often attributed to the same drug class as the patients’ index ADR-related admission. Cancer was a major risk factor for ADR-related re-hospitalization within 90 days; other factors included heart failure and increasing age.

## 1. Introduction

The World Health Organization (WHO) defines an adverse drug reaction (ADR) as “any response to a drug which is noxious and unintended, and which occurs at doses normally used in man for prophylaxis, diagnosis or therapy of disease, or for the modification of physiological function” [1]. ADRs cause significant morbidity and mortality, and impose a substantial burden on patients and health systems internationally [2,3]. One systematic review reported that the median prevalence of hospital admissions due to ADRs in adult patients was 7%, based on 21 included studies [3]. Elderly patients are at a higher risk; those aged ≥75 years exhibit a more than four-fold increased risk of ADR-related admissions compared with patients aged 55–64 years [4]. According to an Australian study, 18.9% of unplanned admissions to medical wards of patients aged ≥65 years were ADR-related [5]. An estimated 23% of hospital readmissions within 30 days of acute discharge are attributed to ADRs, and 2.4% of all discharged patients are readmitted to hospital with an ADR within this timeframe [6].

The incidence of ADRs has increased over the past decade, and this upward trajectory is expected to continue in parallel with aging populations, the increased life expectancy of comorbid patients, and the escalating use of medicines [2,7]. A growing body of evidence suggests that patients who have experienced an ADR are at increased risk of a subsequent ADR, exacerbating the problem. By 12 months post-discharge from ADR-related admission, between 8.7 and 13.4% of patients are readmitted to hospital with a subsequent ADR [8,9]. Nevertheless, there are some gaps in our knowledge regarding the risk of ADR-related admissions. There is a paucity of research investigating whether the relative risk of subsequent ADR-related admission is higher after discharge from ADR- compared to non-ADR-related acute admission. Furthermore, the risk associated with previous ADR-related admission has not been explored beyond 12 months post-discharge and, therefore, it is not known whether the risk abates after this time.

The aim of this study was to investigate the incidence and risk of post-index ADR-related hospital admission within 30, 90, and 365 days of discharge for patient cohorts with ADR- and non-ADR-related admissions. The risk of post-index ADR-related admission in these cohorts for up to five years after initial discharge was compared. The secondary aims were to determine the risk factors for post-index ADR-related admission within 90 days of discharge, and to assess whether index and post-index ADR-related admissions in the same individual were often associated with the same ADR code.

## 2. Materials and Methods

### 2.1. Study Design and Setting

This retrospective cohort study was conducted in Tasmania, an island state of Australia with approximately 390,000 adult residents and served by four major public hospitals [10].

### 2.2. Data Source

Admitted Patient Care National Minimum Dataset (APC-NMDS) records pertaining to all public hospital admissions in Tasmania between January 2011 and May 2017 were extracted and linked with mortality records. The APC-NMDS comprises data mandated for collection by Australian public hospitals, including patient demographics and any diagnoses associated with the admission based on the International Statistical Classification of Diseases and Related Health Problems, tenth edition, Australian Modification (ICD-10-AM) [11]. ICD-10-AM is a classification system developed by the WHO and modified for an Australian context, which contains codes for diagnoses, symptoms, and medical procedures [11].

### 2.3. Eligible Patients and Acute Admissions

Eligible patients were defined as Tasmanian residents aged ≥18 years with an acute hospital admission between January 2011 and December 2015. Acute admissions (defined as overnight medical and surgical admissions, excluding obstetrics, dialysis, and day admissions) were included; records pertaining to all other admission types were deleted from the dataset. Inter-hospital patient transfers or admissions downgraded to non-acute care prior to discharge were combined into single admissions prior to analysis.

The dataset was then screened to identify eligible patients and their acute admissions during the study period. Each patient’s first acute admission during this period was defined as their index admission. Data pertaining to eligible patients’ acute admissions up to 31 May 2017 were captured, facilitating a minimum follow-up of 17 months per patient.

### 2.4. ADR-Related Admissions

ICD-10-AM external cause codes between Y40 and Y59 align with the WHO ADR definition, and are commonly used for ADR identification [12,13]. These codes include instances in which the correct drug, properly administered in therapeutic or prophylactic dosage, is the cause of the experience of adverse effects by patients during their surgical or medical care, excluding accidental overdose, the administration of the wrong drug, instances in which the wrong drug is taken in error, and accidents involving the technique through which drugs are administered [14]. The utility of administrative data to identify ADRs was further developed by Du et al. with the identification of an expanded set of diagnosis-based ICD-10-AM codes that have a high/very high probability of being ADR-related (Supplementary Material Table S1) [15]. Admissions were classified as ADR-related when at least one external cause or diagnosis-based code was associated with the admission according to the APC-NMDS record. Only ADRs that manifested in the community were included; if the ADR developed during the hospital episode, the admission was not classified as ADR-related.

### 2.5. Data Analysis

Patients were assigned to one of two cohorts based on whether their index admission was ADR- or non-ADR-related. Descriptive statistics were used to summarize the baseline characteristics of patients in each cohort. The Charlson Comorbidity Index (CCI) was computed as per Quan et al.’s methodology [16]. Only comorbidities associated with the patients’ index admissions were used to calculate the Charlson Comorbidity Index (CCI).

The proportion of patients in each cohort with a post-index ADR-related admission within 30, 90, and 365 days of discharge and at the end of follow-up was reported. From 2011–2015, the number of ADR-related admissions and the proportion of acute admissions that were ADR-related were determined.

Survival analysis was conducted to determine the probability of patients from each cohort experiencing a post-index ADR-related admission within 5 years of their index discharge. Patients were censored at death or end of follow-up. Risks and relative risks of experiencing post-index ADR-related admissions within various timeframes were derived from survival curves (Appendix A).

A matched subset of patients was created using propensity score analysis. Propensity scores for each patient were estimated using logistic regression. After calculating the propensity scores, 1:1 matching was conducted using the nearest-neighbor method, which matched patients in the ADR-related index admission cohort with patients in the non-ADR-related index admission cohort with the closest propensity score. Supplementary survival analysis was then undertaken using this matched subset of patients to assess (1) the risk of non-ADR-related acute admission after the index discharge and (2) the risk of ADR-related admission after the index discharge.

Post-index ADR-related admissions associated with the same ADR code as the patients’ index ADR-related admissions were defined as repeats. The proportion of repeat post-index ADR-related admissions was calculated.

Risk factors for post-index ADR-related admission within 90 days of discharge were identified using backward stepwise logistic regression. A significance level of ≤0.05 was used as the threshold for inclusion of variables in the final model. Data analysis was conducted using RStudio 1.0.153 (RStudio Team (2016). RStudio: Integrated Development for R. RStudio, PBC, Boston, MA, USA, URL http://www.rstudio.com/, downloaded on 31 August 2017) and the additional R packages listed in Supplementary Material Table S2. Patients who died during their index admission or within 90 days of discharge were excluded from this analysis.

## 3. Results

In total, 91,550 adult patients with an acute hospital admission between January 2011 and December 2015 were included in the study. This represents approximately a quarter of all Tasmanian adults [10]. Patients’ baseline characteristics are shown in Table 1. Patients with an ADR-related index admission comprised 3.1% (2843/91,550) of the study population, and tended to be older and female.

An external cause code was recorded for 72.5% (2060/2843) of ADR-related index admissions. The most common drug categories implicated in these admissions were antihypertensives (347/2843; 12.2%), anticoagulants (289; 10.2%), antineoplastic agents (229; 8.1%), opioids (221; 7.8%), systemic antibiotics (160; 5.6%), diuretics (156; 5.5%), glucocorticoids and their synthetic analogues (113; 4.0%), and antidepressants (111; 3.9%) (Supplementary Material Table S3).

**Figure 1:**
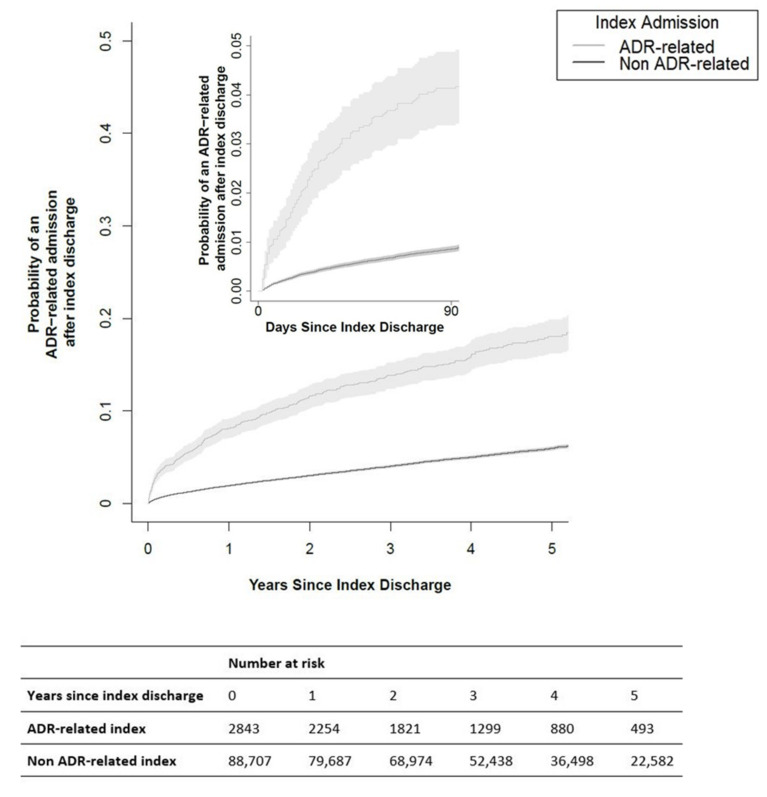
Probability of post-index ADR-related admission for patients with ADR compared to non-ADR-related index admissions.

The proportion of patients with an ADR-related index admission who experienced a post-index ADR-related admission by 30 days after discharge was 2.6% (73/2843), compared to the 0.4% (384/88,707) of patients with a non-ADR-related index admission (*p* < 0.001). By 90 days after discharge, 3.9% (112/2843) and 0.8% (735/88,707) of the patients with ADR and non-ADR-related index admissions, respectively, had experienced a post-index ADR-related admission (*p* < 0.001). The post-index ADR-related admission rates at 365 days after discharge were 7.5% (200/2843) and 1.8% (1569/88,707) for each cohort (*p* < 0.001). Patients were followed up until May 2017 (for a median of 3 years 7 months), by which time 4.9% (4455/91,550) had experienced a post-index ADR-related admission. In the cohort of patients with an ADR-related index admission, 390 (13.7%) experienced a post-index ADR-related admission by the end of the study, of which 209 (53.6%) were repeat events with the same ADR code recorded as the index admission. The calculation of the risks and relative risks from the survival curves (Appendix A) showed that the patients with an ADR-related index admission had a significantly increased risk of post-index ADR-related admission within 30 days (0.0267 vs. 0.00446, RR 5.99, 95% CI 5.34–6.72, *p* < 0.001), 90 days (0.0413 vs. 0.00859, RR 4.81, 95% CI 4.39–5.27, *p* < 0.001), and 365 days (0.0815 vs. 0.0193, RR 4.21, 95% CI 3.95–4.50, *p* < 0.001) after initial discharge (Figure 1).

**Figure 2:**
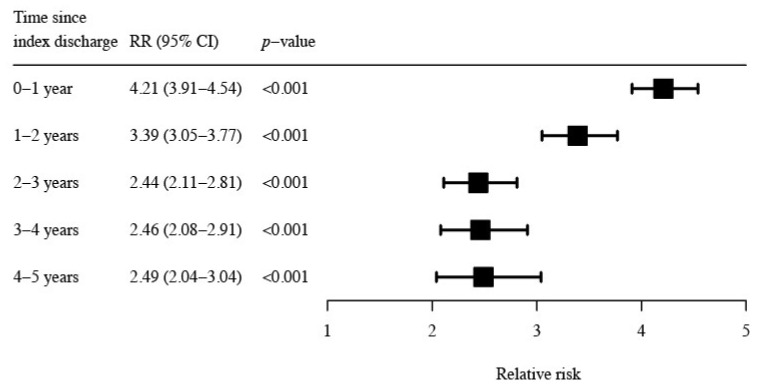
Relative risk of a post-index ADR-related admission for patients with ADR compared to non-ADR-related index admissions. Bars represent 95% confidence intervals.

Additional survival analyses undertaken for each year of follow-up showed that the patients with an index ADR-related admission had a significantly increased risk of a post-index ADR-related admission that persisted for up to 5 years. The relative risk of ADR-related admission in the second year of follow-up was 3.39 (95% CI 3.05–3.77, *p* < 0.001), and a more than two-fold increased risk was observed in each of the following three years (*p* < 0.001) (Figure 2).

The use of propensity matching achieved a balance of all the matched variables based on a threshold of 0.1 for the standardized mean difference (Table 2). In total, 2843 pairs (5686 patients) were successfully matched and included in the supplementary survival analysis. ADR-related index admissions were associated with a significantly increased risk of subsequent ADR-related admission within 90 days (RR 2.40, 95% CI 2.06–2.80, *p* < 0.0001) and 365 days (RR 2.14, 95% CI 1.92–2.38, *p* < 0.0001) of initial discharge. There was no significant difference in the risk of acute non-ADR-related admission within 90 days (RR 1.08, 95% CI 1.02–1.15, *p* = 0.166) or 365 days (RR 1.02, 95% CI 0.99–1.06, *p* = 0.514) of initial discharge.

Logistic regression was used to identify the risk factors for experiencing a post-index ADR-related admission within 90 days of discharge from an acute admission (Table 3). No strong collinearity between predictor variables was detected (all the variance inflation factors were less than 2). A diagnosis of cancer (OR 7.99, 95% CI 6.73–9.47, *p* < 0.001) and an ADR-related index admission (OR 3.95, 95% CI 3.15–4.89, *p* < 0.001) were the most strongly associated with an ADR-related admission within 90 days of discharge. Other associated factors were cardiovascular disease (heart failure, peripheral vascular disease or myocardial infarction), diabetes with complications, mild liver disease, rheumatic disease, and increasing age. The medical patients were at greater risk of a post-index ADR-related admission than the surgical patients.

## 4. Discussion

This retrospective study of 91,550 patients, comprising almost a quarter of the adult population of Tasmania, provides important insights into the burden of ADR-related admissions on the health system [10]. Approximately 4% of all acute hospital admissions between 2011 and 2015 were ADR-related, based on administrative data. While the first 30 days after discharge were found to be particularly hazardous, the study’s major finding was the persistence of ADR-related admission risk in patients with a prior ADR-related admission. Patients with an index ADR-related admission exhibited more than a four-fold increased risk of experiencing a post-index ADR-related admission in their first year of follow-up. A significantly increased risk was also observed during each subsequent year. These novel findings demonstrated that the patients’ ADR susceptibility persisted for at least 5 years, substantially longer than previously reported [18].

The risk factors for experiencing multiple hospital admissions associated with or extended by the development of an ADR were explored previously [12]. However, our study was designed to comprehensively assess ADR-related admission risk. With our large sample size and the inclusion of patients with ADR and non-ADR-related index admissions, we addressed gaps in the literature regarding the excess risk associated with a previous ADR-related admission in the short and long term. Furthermore, our study focused only on ADRs leading to hospital admission. We ascertained that the ADR-related admission history remained a significant predictor of ADR-related admission risk within 90 days of discharge after adjusting for clinical and demographic characteristics. The survival analysis undertaken on the propensity-matched subset, in which the known ADR risk factors, including age and CCI, were evenly distributed, showed that an ADR-related index admission was associated with a more than two-fold increase in ADR-related admission risk within 90 and 365 days of discharge. There was no significant difference in the risk of experiencing an acute non-ADR-related admission during these periods. These findings suggest that a subset of patients may be predisposed to developing ADRs due to risk factors that are distinct from their underlying clinical profile and the social determinants of health that may contribute to their overall readmission risk.

This is an important finding in the context of ADR-related admission risk. It suggests that a patient’s ADR susceptibility is akin to a chronic condition, necessitating ongoing precautions and judicious prescriptions to mitigate ADR risk over the long term. Our findings are also aligned with the work of Stevenson et al., who proposed that medication-related harm be considered a ‘geriatric syndrome’; recognition of this may re-orientate practice to better address the confluence of patient characteristics, medication regimens, and clinical care, which can lead to medication-related harm in elderly patients [19].

Whilst ADR history was a significant risk factor for ADR-related admissions, the elucidation of additional risk factors has important clinical implications. Interventions designed to improve transitions of care, particularly medication safety at discharge, can be expensive and resource-intensive, and, therefore, there is an imperative to target these services to higher-risk patients, who would be expected to derive the most benefit [20]. Hence, this study aimed to identify the risk factors for experiencing ADR-related admission after acute discharge. Our study was sufficiently powered to identify a number of comorbidities associated with significantly increased odds of ADR-related admission within 90 days of discharge from an acute hospital episode; cancer, cardiovascular disease, diabetes with complications, mild liver disease, and rheumatic disease were identified as risk factors in our adjusted multivariable model.

Increasing age is commonly reported as a risk factor for ADR development [4]. Here, the risk peaked in those aged 65–74 years, then tapered slightly in the cohort aged ≥75 years. In the absence of clear biological markers of age, studies of ADRs in the elderly usually include patients aged ≥65 years [21]. However, a novel finding in our study was that even after adjusting for other variables, the patients aged between 45 and 65 years demonstrated significantly increased odds of ADR-related admission within 90 days of discharge compared with the younger patients.

It has been estimated that one in two ADR-related hospital admissions in adult patients is preventable [22]. An Australian study found that this rate increased to 88.5% of ADR-related hospital admissions in patients aged ≥65 years [9]. Although we were not able to determine the causality or preventability of ADR-related admissions, it is important to note that a significant number in our study were also likely to have been preventable, and, moreover, that steps can be taken to mitigate ADR risk [23]. This is especially pertinent for the significant proportion of patients whose post-index ADR-related admission was a repeat event. The medical patients’ significantly increased odds of ADR-related admission within 90 days of discharge may reflect their different clinical profiles; medical patients are older, take more medications, and are subject to more medication changes during their admission compared to surgical patients [24].

A limitation of this work was its retrospective design, which utilized administrative data that detects ADR-related admissions at a relatively low rate. It has been estimated that between 18 and 35% of ADR-related admissions captured prospectively can be identified via administrative data sources, although these rates can be affected by the ICD codes used [13,25,26]. In an Australian study, almost one in six (15%) medical admissions of patients aged ≥65 were classified as ADR-related, based on a prospective review, compared to 2.7% of the same patient cohort using administrative coding [26]. Furthermore, using administrative data, it was not possible to determine the circumstances in which the ADRs developed, judge the appropriateness of the patients’ medication regimens, or assess the impact of polypharmacy on ADR risk.

The strengths of this study included the capturing of ADR-related admissions over the long term and the large sample size, which facilitated the robust statistical analysis. Due to its relative isolation, Tasmania is an ideal location for tracking all hospital admissions over time. It would be reasonable to expect that most eligible patients’ hospital admissions were captured in our dataset, given that they occurred within the Tasmanian health system.

## 5. Conclusions

In conclusion, patients with a prior ADR-related admission are at a significantly increased risk of future ADR-related admissions in the short and long term. These admissions are often related to the same ADR based on ICD-10-AM codes. Whilst efforts to bridge the gap between the hospital and community during transitions of care should remain a priority for patients and healthcare providers alike, our results show that such endeavors should extend beyond the immediate post-discharge period. To optimize prescription decisions and medication management strategies, healthcare professionals should be made aware of any prior ADR-related hospital admissions experienced by their patients. Future research should explore the processes of care that may contribute to ADR-related admission risk after hospital discharge and identify opportunities for improvements in patient care after ADR-related admission.

## Figures and Tables

**Table 1:** Patient characteristics at baseline ^a^.

Characteristic	ADR-Related Index Admission (*n* = 2843) % (*n*)	Non-ADR-Related Index Admission (*n* = 88,707) % (*n*)	*p*-Value
Median length of follow-up, months (interquartile range)	38 (22–57)	43 (27–61)	<0.001
Age (years)			<0.001
<45	21.5% (611)	30.4% (26,926)	
≥45 to <65	25.2% (716)	29.9% (26,519)	
≥65 to <75	18.7% (532)	17.1% (15,124)	
≥75	34.6% (984)	22.7% (20,138)	
Sex (male)	45.5% (1294)	49.5% (43,873)	<0.001
Socio-Economic Indexes for Areas (SEIFA) ^b^			0.29
Quantile 1	21.6% (614)	19.5% (17,298)	
Quantile 2	20.7% (589)	20.8% (18,451)	
Quantile 3	25.2% (716)	27.0% (23,951)	
Quantile 4	32.5% (924)	32.7% (29,007)	
Australian-born	86.0% (2445)	85.8% (76,101)	>0.99
Admission type			
Medical	90.5% (2573)	64.3% (57,062)	<0.001
Surgical	9.5% (270)	35.7% (31,645)	<0.001
Median length of stay (days) (interquartile range)	4 (2–9)	3 (1–7)	<0.001
Charlson Comorbidity Index (CCI)			<0.001
0	56.0% (1592)	68.8% (61,033)	
1–2	27.4% (779)	22.2% (19,696)	
≥3	16.7% (475)	9.0% (7978)	
Mild liver disease	2.5% (71)	0.9% (761)	<0.001
Severe liver disease	0.7% (20)	0.3% (246)	0.01
Renal impairment	7.6% (216)	2.5% (2241)	<0.001
Chronic pulmonary disease	5.7% (162)	4.2% (3737)	<0.001
Peripheral vascular disease	1.1% (31)	1.0% (842)	0.97
Diabetes	9.9% (281)	6.2% (5453)	<0.001
Diabetes with complications	8.4% (239)	4.4% (3914)	<0.001
Dementia	7.8% (222)	3.7% (3310)	<0.001
Rheumatologic disease	1.9% (54)	0.5% (442)	<0.001
Cerebrovascular disease	2.9% (82)	4.1% (3631)	0.03
Heart failure	6.6% (188)	3.5% (3100)	<0.001
Myocardial infarction	1.9% (54)	3.2% (2871)	<0.001
Peptic ulcer	1.0% (28)	0.2% (168)	<0.001
Paraplegia	2.3% (65)	2.1% (2241)	0.99
Cancer	8.8% (250)	6.5% (5766)	<0.001

^a^ The ICD codes used to define the medical conditions included in Table 1 were based on Quan et al. [16]. ^b^ SEIFA, Socio-Economic Indexes for Areas, developed by the Australian Bureau of Statistics to rank areas in Australia according to relative socio-economic advantage and disadvantage [17]. Quantile 1 represents the highest level of socioeconomic advantage.

**Table 2:** Patient characteristics at baseline in propensity-matched subset.

	Matched Subset		Standardized Mean Difference	
Characteristic	ADR-Related Index Admission (*n* = 2843) % (*n*)	Non-ADR-Related Index Admission (*n* = 2843) % (*n*)	Full Dataset (before Propensity Score Matching)	Matched Subset
Age (years)				
<45	21.5% (612)	21.2% (603)	−0.215	0.008
≥45 to <65	25.2% (716)	25.2% (716)	−0.109	0.000
≥65 to <75	18.7% (531)	18.0% (513)	0.042	0.016
≥75	34.6% (984)	35.6% (1011)	0.250	−0.020
Sex (male)	45.5% (1293)	45.0% (1279)	−0.080	0.010
Socio-Economic Indexes for Areas (SEIFA)				
Quantile 1	21.6% (615)	22.8% (647)	0.051	−0.027
Quantile 2	20.6% (587)	19.8% (564)	−0.003	0.020
Quantile 3	25.2% (716)	25.2% (717)	−0.042	−0.001
Quantile 4	32.5% (925)	32.2% (915)	−0.003	0.008
Australian-born	86.0% (2445)	84.6% (2404)	0.006	0.042
Admission type			0.893	−0.041
Medical	90.5% (2573)	91.7% (2607)	−0.893	0.041
Surgical	9.5% (270)	8.3% (236)		
Median length of stay (days) (interquartile range)	4 (2–9)	4 (2–8)	0.142	0.049
Charlson Comorbidity Index (CCI)				
0	56.0% (1591)	57.0% (1621)	−0.259	−0.021
1–2	27.4% (778)	27.2% (772)	0.116	0.005
≥3	16.7% (474)	15.8% (450)	0.206	0.023
Mild liver disease	2.5% (70)	2.4% (69)	0.104	0.002
Severe liver disease	0.7% (19)	0.8% (22)	0.048	−0.013
Renal impairment	7.6% (215)	7.2% (205)	0.190	0.013
Chronic pulmonary disease	5.7% (163)	5.0% (142)	0.065	0.032
Peripheral vascular disease	1.1% (31)	1.1% (30)	0.014	0.003
Diabetes	9.8% (280)	8.6% (244)	0.124	0.042
Diabetes with complications	8.4% (238)	7.2% (205)	0.143	0.042
Dementia	7.8% (222)	7.4% (211)	0.152	0.014
Rheumatologic disease	1.9% (55)	1.5% (44)	0.104	0.028
Cerebrovascular disease	2.9% (82)	3.0% (85)	−0.072	−0.006
Heart failure	6.6% (188)	4.7% (135)	0.125	0.075
Myocardial infarction	1.9% (53)	1.6% (46)	−0.101	0.018
Peptic ulcer	1.0% (28)	0.7% (20)	0.081	0.028
Paraplegia	2.3% (65)	1.9% (55)	0.011	0.024
Cancer	8.8% (250)	9.7% (275)	0.082	−0.031

**Table 3:** Risk factors for a post-index ADR-related admission within 90 days of discharge ^a,b^.

	Full Model		Final Model ^c^	
	Odds Ratio	*p*-Value	Odds Ratio (95% CI)	*p*-Value
ADR-related index	3.38	<0.001	3.47 (2.76–4.32)	<0.001
Age (reference group < 45) (years)				
≥45 <65	1.35	0.007	1.35 (1.08–1.68)	0.008
≥65 <75	1.78	<0.001	1.76 (1.40–2.22)	<0.001
≥75	1.52	<0.001	1.53 (1.22–1.92)	<0.001
Sex (male)	0.90	0.14		
History of myocardial infarction	1.79	0.001	1.76 (1.23–2.46)	0.001
Heart failure	2.56	<0.001	2.73 (2.09–3.51)	<0.001
Peripheral vascular disease	2.07	0.01	2.06 (1.11–3.48)	0.012
Cerebrovascular disease	0.76	0.31		
Chronic pulmonary disease	1.23	0.18		
Dementia	0.94	0.76		
Rheumatologic disease	1.91	0.05	1.97 (0.97–3.55)	0.039
Peptic ulcer	0.99	>0.99		
Diabetes	1.12	0.40		
Diabetes with complications	1.28	0.10	1.37 (1.03–1.79)	0.024
Paraplegia	0.70	0.34		
Renal disease	1.22	0.28		
Cancer	7.97	<0.001	7.99 (6.73–9.47)	<0.001
Mild liver disease	2.75	<0.001	2.37 (1.36–3.82)	<0.001
Severe liver disease	0.24	0.17		
Born overseas	0.95	0.65		
Admission type (reference group surgical)	2.13	<0.001	2.11 (1.78–2.53)	<0.001
Length of hospital stay (days)	1.00	0.07		
SEIFA ^d^		0.20		

^a^ All the variables included in these models pertain to the patients’ index-admission records. ^b^ Due to non-proportional hazards over time, Cox proportional hazard analysis was not an appropriate technique for this dataset. Therefore, logistic regression was used to identify risk factors for admission at a single time point. ^c^ The final model was constructed by successively removing predictor variables with the largest *p*-values (>0.05) until all remaining predictors were significant (*p* ≤ 0.05). Variables were removed from the model in the following order: peptic ulcer, dementia, born overseas, diabetes, paraplegia, renal disease, SEIFA, chronic pulmonary disease, severe liver disease, sex, length of stay, cerebrovascular disease. ^d^ SEIFA, Socio-Economic Indexes for Areas, developed by the Australian Bureau of Statistics to rank areas in Australia according to relative socio-economic advantage and disadvantage [17]. A *p*-value for the four SEIFA quartiles was determined using an ANOVA test to compare the full model with and without SEIFA, included as a categorical variable.

**Figure A1:**
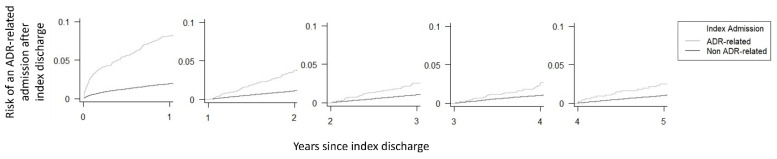
Risk of an ADR-related admission after index discharge for patients with ADR compared to non-ADR-related index admissions.

## Data Availability

The data analyzed in this study were obtained from the Tasmanian Government Department of Health. Human Research Ethics Committee approval is required to access this dataset. Requests to access this dataset should be directed to the Tasmanian Department of Health.

## Supplementary Materials

The following supporting information can be downloaded at: https://www.mdpi.com/article/10.3390/ijerph19095585/s1. Table S1: Du et al. ADR Codes. Table S2: R Packages used for data analyses. Table S3: External cause codes with frequency ≥5 at index.

## Abbreviations

Admitted patient care national minimum dataset (APC-NMDS), Adverse drug reaction (ADR), Charlson Comorbidity Index (CCI), International Statistical Classification of Diseases and Related Health Problems, tenth edition, Australian Modification (ICD-10-AM), Socio-Economic Indexes for Areas (SEIFA), World Health Organization (WHO).

## Appendix A

The Kaplan Meier survival analysis generated functions *S*(*t*) representing the probability of “survival” (i.e., not having an ADR-related admission) over time. Hence the probability or risk *A*(*t*) of having an ADR-related admission risk within time *t* is

*A(t) = 1 – S(t)*

For each cohort, *A*(*t*) was calculated for times *t* equal to 30, 90 and 365 days after discharge. The relative risk *RR*(*t*) of an ADR-related admission within 30, 90 and 365 days for cohorts of patients with ADR versus non-ADR-related index admissions was then computed by dividing the relevant ADR-related admission risks:

*RR*(*t*) = A(*t*) (ADR-related index)/*A*(*t*) (Non-ADR-related index)

Survival analysis was also conducted for discrete time periods: 1–2, 2–3, 3–4 and 4–5 years after the index discharge. For each period, only patients who had not been censored and had not experienced an ADR-related admission at the beginning of the period were included in these analyses, and relative risks at the end of each period were calculated using the procedure above.

The survival curves generated for 0–1, 1–2, 2–3, 3–4 and 4–5 years after index discharge are illustrated in Figure 1. Note that the survival curve for the first year of follow-up is a reconstruction of Figure 1 from the main text, with the x-axis restricted to 0–1 years.

Confidence intervals (CIs) were calculated using log-minus-log transformations to ensure that the CIs were between 0 and 1 [27].

The supplementary survival analysis undertaken to assess ADR-related admission risk in a matched subset of patients with ADR versus non-ADR-related index admissions also reflected this methodology.

The risk of an acute non-ADR-related admission within 90 days after discharge required similar computations. *S*(*t*) in this analysis represented the probability of “survival” (i.e., of not experiencing an acute non-ADR-related admission) over time. The probability or risk *N* (*t*) of experiencing an acute non-ADR-related admission within time *t* is:

*N(t) = 1 – S(t)*

The relative risk of an acute non-ADR-related admission at 90 days was calculated as per the relative risk formula outlined above.
