# Supplementary material for: Persistence of Adverse Drug Reaction-Related Hospitalization Risk Following Discharge
Source: Int J Environ Res Public Health. 2022 May 4;19(9):5585. doi: 10.3390/ijerph19095585 (PMC9101512; doi:10.3390/ijerph19095585)
Supplement: Supplementary file 1 [file ijerph-19-05585-s001.zip › Supplementary Material Table S1.pdf]

Supplementary Material Table S1. Du et al. ADR Codes.[1]

ICD-10-AM Code	Description
D52.1	Drug-induced folate deficiency anemia
D59.0	Drug-induced autoimmune hemolytic anemia
D59.2	Drug-induced nonautoimmune hemolytic anemia
D61.1	Drug-induced aplastic anemia due to chemotherapy
D64.2	Secondary sideroblastic anemia due to drugs and toxins
D68.3	Hemorrhagic disorder due to circulating anticoagulants
E03.2	Hypothyroidism due to medicaments and other exogenous substances
E06.4	Drug-induced thyroiditis
E16.0	Drug-induced hypoglycemia without coma
E23.1	Drug-induced hypopituitarism
E24.2	Drug-induced Cushing’s syndrome
E27.3	Drug-induced adrenocortical insufficiency
E66.1	Drug-induced obesity
F11	Mental and behavioral disorders due to use of opioids
F13	Mental and behavioral disorders due to use of sedatives or hypnotics
G21.0	Malignant neuroleptic syndrome
G21.1	Other drug-induced secondary parkinsonism
G21.2	Secondary parkinsonism due to other external agents
G24.0	Drug-induced dystonia
G25.1	Drug-induced tremor
G25.4	Drug-induced chorea
G25.6	Drug-induced tics and other tics of organic origin
G44.4	Drug-induced headache not elsewhere classified
G62.0	Drug-induced polyneuropathy
G72.0	Drug-induced myopathy
H26.3	Drug-induced cataract
H40.6	Glaucoma secondary to drugs
I42.7	Cardiomyopathy due to drugs and other external agents
I95.2	Hypotension due to drugs
J70.2	Acute drug-induced interstitial lung disorders
J70.3	Chronic drug-induced interstitial lung disorders
J70.4	Drug-induced interstitial lung disorders (unspecified)
K85.3	Drug-induced pancreatitis
L10.5	Drug-induced pemphigus
L23.3	Allergic contact dermatitis due to drugs in contact with skin
L24.4	Irritant contact dermatitis due to drugs in contact with skin
L25.1	Unspecified contact dermatitis due to drugs in contact with skin
L27	Dermatitis due to substances taken internally
L43.2	Lichenoid drug reaction
L56.0	Drug phototoxic response

ICD-10-AM Code	Description
L56.1	Drug photoallergic response
L64.0	Drug-induced androgenic alopecia
M10.2	Drug-induced gout
M32.0	Drug-induced systemic lupus erythematosus
M34.2	Systemic sclerosis induced by drugs and chemicals
M80.4	Drug-induced osteoporosis with pathological fracture
M81.4	Drug-induced osteoporosis without pathological fracture
M83.5	Other drug-induced osteomalacia in adults
M87.1	Osteonecrosis due to drugs
N14	Analgesic nephropathy
Q86.2	Dysmorphism due to warfarin
R50.2	Drug-induced fever
T78.2	Anaphylactic shock (unspecified)
T78.3	Angioneurotic edema
T78.4	Allergy (unspecified)
T78.8	Other adverse effects not elsewhere classified
T78.9	Adverse effect (unspecified)
T80.1	Vascular complications following infusion transfusion and therapeutic injection
T80.2	Infections following infusion transfusion and therapeutic injection
T80.3	ABO incompatibility reaction
T80.5	Complications following infusion transfusion and therapeutic injection anaphylactic shock due to serum
T80.6	Complications following infusion transfusion and therapeutic injection other serum reactions
T80.8	Other complications following infusion transfusion and therapeutic injection
T80.9	Unspecified complication following infusion transfusion and therapeutic injection
T88.3	Malignant hyperthermia due to anesthesia
T88.6	Anaphylactic shock due to adverse effect of correct drug or medicament properly administered
T88.7	Unspecified adverse event of drug or medicament
Z03.6	Observation for suspected toxic effect from ingested substance

1. Du W, Pearson SA, Buckley NA, et al. Diagnosis-based and external cause-based criteria to identify adverse drug reactions in hospital ICD-coded data: application to an Australia population-based study. Public Health Res Pract. 2017; 27: e2721716.
