# Supplementary material for: Persistence of Adverse Drug Reaction-Related Hospitalization Risk Following Discharge
Source: Int J Environ Res Public Health. 2022 May 4;19(9):5585. doi: 10.3390/ijerph19095585 (PMC9101512; doi:10.3390/ijerph19095585)
Supplement: Supplementary file 1 [file ijerph-19-05585-s001.zip › Supplementary Material Table S2.pdf]

Supplementary Material Table S2. R Packages used for data analysis

Package	Reference
stringr	Hadley Wickham (2019). stringr: Simple, Consistent Wrappers for Common String Operations. R package version 1.4.0. https://CRAN.R-project.org/package=stringr
plyr	Hadley Wickham (2011).The Split-Apply-Combine Strategy for Data Analysis. Journal of Statistical Software, 40(1), 1-29. http://www.jstatsoft.org/v40/i01/.
dplyr	Hadley Wickham, Romain François, Lionel Henry and Kirill Müller (2019).dplyr: A Grammar of Data Manipulation. R package version 0.8.0.1. https://CRAN.R-project.org/package=dplyr
data.table	Matt Dowle and Arun Srinivasan (2019). data.table: Extension of ‘data.frame`. R package version 1.12.2. https://CRAN.R-project.org/package=data.table
tidyr	Hadley Wickham and Lionel Henry (2019). tidyr: Easily Tidy Data with ‘spread()' and 'gather()' Functions. R package version 0.8.3. https://CRAN.R-project.org/package=tidyr
ggplot2	H. Wickham. ggplot2: Elegant Graphics for Data Analysis. Springer-Verlag New York, 2016.
survminer	Alboukadel Kassambara, Marcin Kosinski and Przemyslaw Biecek (2019). survminer: Drawing Survival Curves using 'ggplot2'. R package version 0.4.5. https://CRAN.R-project.org/package=survminer
survival	Therneau T (2015). _A Package for Survival Analysis in S_. version 2.38, https://CRAN.R-project.org/package=survival<br>Terry M. Therneau, Patricia M. Grambsch (2000). Modeling Survival Data: Extending the Cox Model_. Springer, New York. ISBN 0-387-98784-3.
forestplot	Max Gordon and Thomas Lumley (2019). forestplot: Advanced Forest Plot Using 'grid' Graphics. R package version 1.9. https://CRAN.R-project.org/package=forestplot
mctest	Imdad, M. U. & Aslam, M. (2018). mctest: Multicollinearity Diagnostic Measures. https://CRAN.R-project.org/package=mctest, R package version 1.2<br>Imdadullah, M., Aslam, M., and Altaf, S., (2016). mctest: An R Package for Detection of Collinearity among Regressors. The R Journal, 8(2),499—509 https://journal.r-project.org/archive/2016/RJ-2016-062/index.html
MatchIt	Daniel E. Ho, Kosuke Imai, Gary King, Elizabeth A. Stuart (2011). MatchIt: Nonparametric Preprocessing for Parametric Causal Inference. Journal of Statistical Software, Vol. 42, No. 8, pp. 1-28. https://doi.org/10.18637/jss.v042.i08
