# Supplementary material for: Persistence of Adverse Drug Reaction-Related Hospitalization Risk Following Discharge
Source: Int J Environ Res Public Health. 2022 May 4;19(9):5585. doi: 10.3390/ijerph19095585 (PMC9101512; doi:10.3390/ijerph19095585)
Supplement: Supplementary file 1 [file ijerph-19-05585-s001.zip › Supplementary Material Table S3.pdf]

Supplementary Material Table S3. External Cause Codes with Frequency ≥5 at Index [a]

Category	ICD-10-AM codes	Frequency
Antihypertensives	Y517, Y521, Y523, Y524, Y525	347
Anticoagulants	Y442	289
Antineoplastic agents	Y431, Y432, Y433	229
Opioids	Y450	221
Systemic antibiotics (excluding antifungals)	Y400, Y401, Y402, Y403, Y404, Y405, Y406, Y408, 409	160
Diuretics	Y541, Y542, Y543, Y544, Y545	156
Glucocorticoids and synthetic analogues	Y420	113
Antidepressants	Y490, Y491, Y492	111
Antiepileptic and antiparkinsonism agents	Y460, Y461, Y462, Y463, Y464, Y465, Y466, Y467, Y468	82
NSAIDs (excluding salicylates) [b]	Y452, Y453	80
Antidysrhythmic	Y520, Y522	69
Psychotropics (excluding antidepressants)	Y493, Y494, Y495, Y496, Y497, Y498, Y499	66
Antidiabetic agents	Y423	64
Analgesics, antipyretics and anti-inflammatories (excluding NSAIDs and opioids)	Y454, Y455, Y459	38
Anti-infective and antiparasitic agents	Y410, Y411, Y412, Y413, Y414, Y415, Y418, Y419	35
Sedatives, hypnotics and anti-anxiolytics	Y470, Y471, Y472, Y473, Y474, Y475, Y478, Y479	33
Antihyperlipidemic and anti-arteriosclerotic agents	Y526	30
Anesthetic therapeutic gases	Y480, Y481, Y482, Y483, Y484, Y485	26
Antihypertensive AND diuretic (combination)		23
Immunosuppressive agents	Y434	21
Other and unspecified hormones and their synthetic substitutes	Y428	18
Antiplatelets	Y444, Y451	17
Alpha-adrenoreceptor antagonists, not elsewhere classified (excluding ergot alkaloids)	Y516	14
Analgesics, antipyretics and anti-inflammatories (excluding NSAIDs and opioids) AND opioids (combination)	Y454, Y455, Y459	14
Agents affecting calcification	Y547	13
Anti-allergic and anti-emetic drugs	Y430	11
Contraceptives	Y424, Y425	9
Other antacids and anti-gastric-secretion drugs	Y531	9
Agents affecting uric acid metabolism	Y548	8
Thyroid hormones	Y421	7
Other parasympatholytic (anticholinergics and antimuscarinics) and spasmolytic agents, not elsewhere classified	Y513	7
Antidepressant AND antiepileptic and antiparkinsonism agent (combination)		7

Category	ICD-10-AM codes	Frequency
Antineoplastic AND glucocorticoids and synthetic analogues (combination)		7
Antithyroid agent	Y422	6
Androgens and anabolic congeners	Y427	6
Anticholinesterase agents	Y510	6
Peripheral vasodilators and nicotinic acid derivatives	Y527	6
Antidepressant AND psychotropic (combination)		6
Antidysrhythmic AND antihypertensive (combination)		6
Iron preparations and other anti-hypochromic-anemia preparations	Y440	5
Opioid receptor antagonists	Y501	5
Stimulant laxatives	Y532	5
Other laxatives	Y534	5
Anti-asthmatics, not elsewhere classified	Y556	5
Antidepressant AND antihypertensive (combination)		5
Antidepressant AND opioid (combination)		5
Antiepileptic and antiparkinsonism agent AND opioid (combination)		5
Antihypertensive AND NSAID (excluding salicylates) (combination)		5

(a) Categories are not mutually exclusive. An external cause code was recorded for 72.5% (2,060/2,843) of ADR-related index admissions.
(b) Nonsteroidal anti-inflammatory drugs (NSAIDs).
